# Exploring Patients' Acceptance of Mental Health E-services in Morocco: A Quantitative Approach

**DOI:** 10.7759/cureus.76143

**Published:** 2024-12-21

**Authors:** Loubna Khalil, Zineb Serhier, Mohammed Bennani Othmani

**Affiliations:** 1 Clinical Neurosciences and Mental Health Laboratory, Hassan II University, Faculty of Medicine and Pharmacy, Casablanca, MAR

**Keywords:** e-appointment system, e-services, mental health, technology acceptance, unified theory of acceptance and use of technology (utaut), use intention

## Abstract

Background

The transformative potential of technology in addressing mental healthcare challenges is more widely acknowledged in Morocco. The government has taken active measures to address persistent mental health challenges and provide better care by exploring innovative digital solutions. Several e-health services initiatives have been implemented, including electronic health record systems, telemedicine services, e-appointment systems, and mobile health applications. However, the adoption of e-health technologies in Morocco, even for basic services such as the e-appointment system (EAS), remains notably low.

Objective

By assessing the constructs of the Unified Theory of Acceptance and Use of Technology (UTAUT) along with additional variables, including trust in technology and perceived mental health conditions, this study aims to identify key predictors influencing patients' acceptance and use of EAS as a reliable way of accessing mental health support.

Methods

Data were collected through a questionnaire survey administered to mental health patients receiving treatment at the Psychiatry Department of the University Hospital in Casablanca, Morocco. Patients were recruited based on their willingness to participate, while those with cognitive difficulties that impaired their ability to complete the survey were excluded. The survey was conducted over seven months, from January to July 2023, with a total of 200 participants enrolled during their visits to the University Hospital. A logistic regression analysis was performed to identify the predictors of EAS acceptance among mental health patients.

Results

The results indicate low adoption rates of the EAS, with many patients only trying it once before reverting to traditional scheduling methods, such as phone calls or in-person visits. Perceptions show optimism about the benefits of EAS but highlight areas for improvement in social and technical support to enhance acceptance. Performance expectancy, trust in technology, and facilitating conditions were found to be significant predictors of EAS acceptance and use among mental health patients. In contrast, effort expectancy, social influence, and perceived mental health conditions were insignificant and didn't appear to influence EAS acceptance meaningfully.

Conclusion

This study's findings can be used to develop effective strategies that promote the widespread adoption of e-mental health services and ultimately address the barriers to accessing quality mental healthcare.

## Introduction

Mental health issues are widespread and inadequately addressed worldwide [1], representing critical public health challenges. In Morocco, healthcare institutions are making concerted efforts to improve the accessibility, effectiveness, patient experience, and delivery of mental healthcare services. This urgency is underscored by a 2022 report from the Economic, Social, and Environmental Council (CESE), which revealed that 48.9% of people over the age of 15 currently exhibit or have previously displayed signs of mental illness [2]. However, the mental health sector still faces various challenges, including stigma, limited resources, and unequal distribution of services across urban and rural areas.

In the current digital era, advancements in technology have revolutionized several aspects of healthcare delivery [3], including the mental health field, where digital technologies offer significant potential to enhance access and quality of care [4]. In Morocco, the transformative potential of technology in addressing mental healthcare challenges is more widely acknowledged.

Several e-health services initiatives have been introduced, including electronic health record systems, telemedicine services, e-appointment services, and mobile health applications. Notably, in 2015, the Ministry of Health implemented a computerized appointment management system, allowing patients to schedule appointments online with ease. By 2022, the "Mawiidi" platform had facilitated over two million appointments successfully, representing 34% of all the appointments made [5].

In 2018, a national telemedicine program was initiated to improve healthcare access in rural and remote areas, supported by the establishment of a regulatory framework to monitor telemedicine practices [6]. Looking ahead, the Moroccan government plans to set up an "intelligent healthcare system," which will introduce a unified national common patient file. This initiative aims to enhance coordination among healthcare stakeholders, reduce diagnostic delays, prevent inappropriate treatments, and minimize medical errors, further advancing the country's digital health transformation.

Despite concerted government efforts to develop and implement various e-health services, adoption rates remain significantly low [7,8]. This is particularly evident even for basic services like electronic medical appointment scheduling, which prior studies have emphasized for its numerous advantages, including enhanced convenience, reduced wait times, and decreased medical care costs [9]. In developing countries, the adoption of e-health services faces persistent barriers [10]. Factors such as limited infrastructure, financial constraints, digital literacy gaps, and cultural resistance continue to hinder their effective implementation and use [11].

The acceptance and use of e-appointment services can pave the way for the widespread integration of diverse e-health services. Experiencing the convenience and efficiency of online scheduling may encourage patients to adopt other digital tools, such as telemedicine consultations and health education platforms. By investigating the key predictors of the acceptance and use of e-appointment system (EAS), we can gain valuable insights into patients' overall readiness to embrace e-health services. These findings can inform the development of effective strategies to encourage the broader adoption of e-mental health services, ultimately helping to overcome barriers to accessing quality mental healthcare.

Research on the acceptance of EAS for accessing mental healthcare remains limited [12]. To address this gap, this study aims to identify the key predictors of patients' acceptance and use of EAS as a reliable means of obtaining mental health support. Focusing on the EAS offered by a university hospital in Morocco, the study employs the Unified Theory of Acceptance and Use of Technology (UTAUT) framework, a widely recognized framework for understanding the adoption and use of technology. The framework is further enriched with additional variables, including trust in technology and perceived mental health conditions, to explore critical predictors specific to mental healthcare. Participants were primarily recruited from urban areas, aligning with the University Hospital's main service region. The research questions guiding this study were as follows: Do Moroccan mental health patients use EAS in their daily routine? What are Moroccan mental health patients' perceptions of EAS? What are the key predictors of EAS acceptance and use for accessing mental health services among Moroccan patients?

This paper begins by presenting the proposed research model and the methods employed to examine Moroccan patients' perceptions, acceptance, and use of EAS. It then details the results of the study conducted within a mental healthcare context. Finally, the paper concludes with a discussion of the findings, key takeaways, and recommendations for future research.

## Materials and methods

Study design and participants

This study aims to investigate the use and acceptance of EAS among mental health patients using a quantitative research approach. Participants were patients receiving care for mental health concerns at the Psychiatry Department of the University Hospital in Casablanca, Morocco, regardless of their types of mental health disorders. Recruitment was based on the patient's willingness to participate, with the exclusion of those with cognitive impairments that would hinder their ability to complete the survey. The survey was conducted over seven months, from January to July 2023, with a total of 200 participants enrolled during their visits to the University Hospital.

Theoretical framework

Since the 1970s, research investigating the factors affecting technology acceptance and adoption has led to the creation of several models enabling the analysis of variations in technology adoption or rejection. The foundational model was the Technology Acceptance Model (TAM) [13], which considers that perceived usefulness and ease of use are the two primary determinants of technology acceptance and use. Over time, TAM has undergone several adaptations incorporating appropriate variables for each study setting.

To address the limitations of existing models and to propose a better explanation of the technology adoption process, researchers examined eight technology acceptance theories. This led to the proposal of the UTAUT model [14], which categorizes factors influencing individuals' intentions to use technology into four key constructs: effort expectancy, performance expectancy, facilitating conditions, and social influence. These factors are further moderated by individual characteristics and external factors.

Performance expectancy refers to the degree to which patients believe that using the EAS will improve their access to mental healthcare [14]. Patients may perceive the system as a way to reduce waiting times, improve convenience, or facilitate timely mental health support.

Effort expectancy focuses on the perceived ease of use associated with using the EAS [14]. Patients may evaluate how simple and user-friendly the system is, particularly considering literacy levels or digital skills.

Social influence encompasses the extent to which patients perceive that persons who are important to them (family, friends, or healthcare providers) think they should use the EAS [14]. Social support or recommendations from trusted individuals can encourage patients to use the e-appointment system as mentioned in prior studies.

Facilitating conditions refer to the degree to which patients believe that technical and organizational resources are available to support the use of the EAS [14]. Factors such as access to devices, internet connection, or clear instructions play a critical role in the acceptance and use of such systems.

The UTAUT model has been extensively validated through empirical research across diverse contexts, including healthcare [8,15]. Numerous studies have demonstrated the applicability and robustness of this model in predicting technology acceptance and use behaviors, providing evidence of its effectiveness as a theoretical model for understanding human behavior regarding health technology adoption [7,16,17].

Our proposed research model focuses on six key predictors to address the research questions above. These predictors include the four variables of the UTAUT model, such as effort expectancy, performance expectancy, social influence, and facilitating conditions, along with additional variables, including trust in technology and perceived mental health conditions.

Trust in technology encompasses the extent of an individual's belief in the capacity of health technology to fulfill their needs in terms of healthcare [18]. This construct is particularly important in mental health settings, where human interaction plays a significant role. Therapeutic relationships are frequently formed through face-to-face exchanges that offer understanding and personalized support [19]. For mental health patients, trust in technology is based on a platform's ability to consistently meet their emotional and therapeutic demands as well as its functionality.

Perceived mental health conditions refer to participants' self-assessments or beliefs about their mental health, which may differ from clinical diagnoses. These perceptions may affect how patients approach treatment and interact with available resources or support systems, including accepting e-health services. The stigma associated with mental health issues can also discourage patients from using digital services, as they may worry about privacy concerns or judgment [20]. Some patients may perceive digital health tools as impersonal or inadequate, especially if they rely on face-to-face interactions for emotional support.

**Figure 1:**
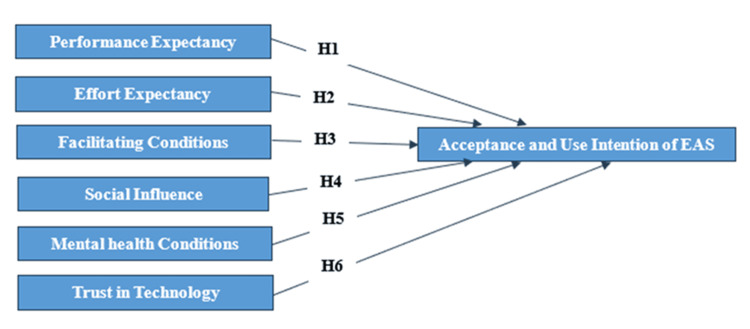
The proposed research model H - hypothesis; EAS - e-appointment system

Based on current research and to identify the effective predictors of accepting and using EAS, each variable was hypothesized to have a direct influence on the intention to use EAS. Figure 1 below illustrates the proposed research model, outlining the hypothesized relationships between the constructs and their impact on the intention to use EAS.

Instruments

The Unified Theory of Acceptance and Use of Technology (UTAUT) served as the basis for the questionnaire survey used to collect data in this study. The survey consisted of twelve questions, divided into eight sections.

The demographics section collected data on participants' age, gender, education level, residence, occupation, and perceived mental health conditions. The EAS use habits section gathered information about participants' preferred methods for scheduling appointments, the frequency and duration of EAS use, the platforms used, and their previous experiences and satisfaction with EAS.

Adapted from UTAUT, the effort expectancy section assessed participants' perceptions of how easy it is to use EAS, while the performance expectancy section evaluated their beliefs about the benefits of EAS in managing their mental health. The social influence section measured the extent to which participants believed that significant individuals, such as friends, family, and healthcare providers, thought they should use EAS.

The facilitating conditions section assessed the degree to which participants felt they had the required resources and support to use EAS effectively. The behavioral intention section measured participants' intention to use EAS in the future. Finally, the trust in technology section was added to reflect its relevance to the study's aims, including questions about participants' trust in EAS and concerns regarding privacy and security.

All items except demographics and EAS use habits were assessed using a five-point Likert scale, with 1 denoting extreme disagreement, 2 - disagreement, 3 - neutrality, 4 - agreement, and 5 - strong agreement. The questionnaire was developed in French and was given out on paper. The questions were translated and explained when the questionnaire was administered directly to participants.

Data analysis

A comprehensive analysis was performed employing descriptive statistics, including means, standard deviations, and percentages, to effectively summarize the sample's demographic features and EAS use habits. Logistic regression analysis was then conducted to explore the predictors of EAS acceptance among mental health patients.

The independent variables comprised effort expectancy, performance expectancy, facilitating conditions, social influence, trust in technology, and perceived mental health conditions, while the dependent variable was the acceptance and use Intention of EAS. Beta coefficients (β), t-values, and p-values were calculated for each construct to evaluate the strength and significance of their relationships.

The overall fit of the regression model was assessed using R-squared (R²) values, which provide insight into the extent to which the independent variables account for the variance in the dependent variable. A p-value found to be lower than 0.05 was considered statistically significant. 

Data analysis was conducted using JAMOVI (The jamovi project, Sydney, Australia) version 2.2.5 and SPSS (IBM, Inc., Armonk, US) version 27. JAMOVI was primarily used for conducting descriptive statistics and reliability testing (Cronbach's alpha) due to its user-friendly interface and minimal computational complexity. SPSS was used for regression analysis, leveraging its versatility for traditional statistical procedures. It provides reliable tests for relationships between the independent variables and the dependent variables. The combination of both software packages allowed for a comprehensive analysis of the data, ensuring the accuracy and reliability of the results.

## Results

Reliability assessment

Content validity was established through expert reviews, ensuring that the questionnaire items aligned with the theoretical constructs and objectives of the study. Cronbach's alpha was computed for each variable to evaluate the internal consistency and reliability of the constructs used in the study. Cronbach's alpha is a widely used statistical test to measure the extent to which items within a given construct are consistent in their measurement. A higher value of Cronbach's alpha (typically above 0.70) indicates that the items within the construct reliably measure the underlying concept.

Each construct was assessed individually based on the items in the survey that were designed to measure specific aspects of the acceptance of the EAS, such as use intention, performance expectancy, effort expectancy, social influence, facilitating conditions, and trust.

Cronbach's alpha was computed for each construct, assessing whether the items within each scale were consistent and measuring the same underlying predictor. All variables exhibited strong internal consistency, as reflected by their Cronbach's alpha values exceeding 0.7. These results confirm the reliability of the questionnaire and support its suitability for measuring the intended constructs. The detailed reliability scores are presented in Table 1.

**Table 1:** The Cronbach’s α value of the constructs EAS - e-appointment system

Constructs	Items	Cronbach's α
Use intention (UI) of EAS	UI1. I intend to use EAS if it is available from my doctor.	0.97
	UI2. I intend to use the EAS in the future if I worry about my mental health.	
Performance expectancy (PEX)	PEX1. Using online services to book a mental health care appointment is more convenient than using the phone or visiting in person.	0.95
	PEX2. Scheduling mental health care appointments online would help me save time.	
	PEX3. Scheduling mental health care appointments online will make it easier and cheaper for me to manage medical appointments.	
	PEX4. Using EAS will enable me to choose a consultation time at my convenience, regardless of the medical facility's hours.	
	PEX5. Overall, I would find online appointment scheduling for mental health care quite beneficial.	
Effort expectancy (EEX)	EEX1. The EAS would be user-friendly for me.	0.94
	EEX2. It's easy for me to learn how to use the EAS.	
	EEX3.It wouldn't take long for me to become proficient with the EAS.	
Social influence (SIN)	SIN1. My Family and friends recommend using online medical appointment booking services.	0.7
	SIN2. If I didn't use the EAS, I would feel out of the loop.	
Facilitating conditions (FCO)	FCO1. I possess the necessary equipment and reliable internet to use the EAS.	0.76
	FCO2. I can ask for help using the EAS from a family member, friend, or a cyber cafe.	
	FCO3. I am confident in my ability to use online scheduling services for mental health care.	
	FCO4. I possess the knowledge and skills required to effectively use the EAS.	
Trust (TRU)	TRU1. I am confident in the reliability of the EAS for managing patient appointments.	0.93
	TRU2. I am confident that my online appointment requests will be processed and not ignored.	
	TRU3. I am confident that the internet offers the security needed when using EAS.	

Demographic characteristics of the sample

Out of the 200 patients initially recruited, 160 completed the survey, resulting in an 80% response rate. The demographic characteristics of the participants, as summarized in Table 2, show that 82 respondents (51.2%) were female and 78 respondents (48.8%) were male, with an average age of 41 years. This provides a balanced representation of gender among the study participants. The largest occupational group comprised homemakers, and the majority of participants reported having attained a university or college-level education. Participants were predominantly from urban areas, reflecting the University Hospital's primary service region. The majority, 125 respondents (78.1%), resided in Casablanca, the largest city in Morocco, while 35 participants (21.9%) came from smaller urban centers. The findings also reveal a significant mental health burden among the participants, with 111 individuals (69.4%) perceiving their mental health as being in poor condition.

**Table 2:** Sample's demographic characteristics

Variables	n (%)
Age (years), mean (SD)	41 (3.13)
Gender	
Female	82 (51.2)
Male	78 (48.8)
Occupation	
Student	25 (15.6)
Employee	30 (18.8)
Freelance professional	34 (21.3)
Retired	10 (6.3)
Job seeker	22 (13.8)
Homemaker	39 (24.4)
Education	
Primary	20 (12.5)
Middle school	32 (20)
High school	32 (20)
University/College	56 (35)
Illiterate	20 (12.5)
Residence city	
Casablanca	125 (78.1)
Other cities	35 (21.9)
Perceived mental health conditions	
Excellent	2 (1.2)
Very good	2 (1.2)
Good	5 (3.1)
Fair	40 (25)
Poor	111 (69.4)

Use habits of EAS

The results indicate low adoption rates, with many patients only trying the EAS once before reverting to traditional scheduling methods, such as phone calls or in-person visits. Among those who have used the EAS, most have only engaged with it a single time. Interestingly, more than half of the EAS users (15 out of 27, or 55.6%) began using the service after the COVID-19 pandemic. Satisfaction levels with EAS have been mixed, reflecting varying experiences among users. Table 3 outlines the detailed use habits of EAS.

**Table 3:** Use habits of EAS EAS - e-appointment system

Variables	n (%)
Method of scheduling appointments	
Traditional way (by phone or in person)	133 (83.12)
Online appointments services	27 (16.88)
Main platform used	
University hospital platform	15 (55.6)
Health Ministry platform	12 (44.4)
Frequency of using EAS	
One time	15 (55.6)
Between 2 and 5 times	10 (37.04)
More than 5 times	2 (7.4)
Period of using EAS	
Before the pandemic crisis (COVID-19)	7 (25.9)
During the pandemic crisis (COVID-19)	5 (18.5)
After the pandemic crisis (COVID-19)	15 (55.6)
Use satisfaction with EAS	
Excellent	3 (11.1)
Very good	5 (18.5)
Good	10 (37.04)
Fair	5 (18.5)
Poor	4 (14.81)

Perceptions and key predictors of EAS acceptance and use

The findings reveal varying perceptions and attitudes toward using EAS. Participants generally perceive EAS as useful, with a high mean score for performance expectancy, indicating a belief in its ability to enhance their healthcare experience. The mean scores for effort expectancy and facilitating conditions suggest moderate perceptions of ease of use and availability of support, respectively. However, social influence scored relatively low, indicating that respondents do not experience significant social pressure or encouragement to use EAS. Table 4 below provides a detailed summary of these findings.

**Table 4:** Perceptions and key predictors of EAS use * significant at p<0.05; ** significant at p<0.01 EAS - e-appointment system; PEX - performance expectancy; EEX - effort expectancy; SIN - social influence; TRU - trust in technology; FCO - facilitating conditions; PMHC - perceived mental health conditions

Variables	Mean	Std.	β	E.S	Wald	Sig.	Exp(β)
Intercept			-15.57	3.50	19.84	0.00**	0.00
PEX	4.21	1.004	2.84	0.67	17.93	0.00**	17.20
EEX	3,56	1.159	-0.31	0.38	0.69	0.41	0.73
SIN	2.30	1.086	0.08	0.33	0.06	0.81	1.08
TRU	3.83	1.019	1.01	0.33	9.25	0.002**	2.74
FCO	3.60	1.139	0.84	0.39	4.69	0.03*	2.31
PMHC	1.40	0.720	0.04	0.68	0.00	0.95	1.04

The binary logistic regression analysis identified several key predictors influencing the acceptance and use of EAS among mental health patients. Performance expectancy emerged as the most significant predictor, with a highly positive influence on the intention to use EAS (β=2.84, p<0.001). For every one-unit increase in performance expectancy, the odds of intending to use EAS increased by a factor of 17.2, suggesting a strong association between high-performance expectancy and the use intention of EAS.

Trust in technology was also a significant predictor (β=1.01, p=0.002). The intention to use EAS increased by 2.7 times for every unit increase in trust in technology, showing a moderate positive effect of trust on the intention to use EAS.

Facilitating conditions were statistically significant (β=0.84, p=0.03), with better-facilitating conditions increasing the likelihood of using EAS. Specifically, a one-unit improvement in facilitating conditions increased the odds by 2.31, suggesting that sufficient support and resources positively impact the acceptance and use.

In contrast, effort expectancy and social influence were not statistically significant predictors of EAS acceptance and use (p>0.05). Although the odds ratio for effort expectancy was less than 1 (0.733), indicating a potential decrease in the likelihood of accepting EAS as effort expectancy increases, this relationship was not statistically significant. Similarly, the odds ratio for social influence was close to 1, suggesting no meaningful effect on the intention to use EAS.

Perceived mental health conditions had no direct effect on the intention to use EAS, as indicated by the non-significant results (β=0.04, p>0.05), suggesting that patients' self-perceptions of their mental health did not significantly influence their likelihood of adopting the service.

The results strongly support the hypotheses that performance expectancy and trust in technology positively influence patients' intention to use EAS. The hypothesis regarding facilitating conditions was partially supported. Conversely, the hypotheses that effort expectancy, social influence, and perceived mental health conditions positively influence the intention to use EAS were not supported.

**Figure 2:**
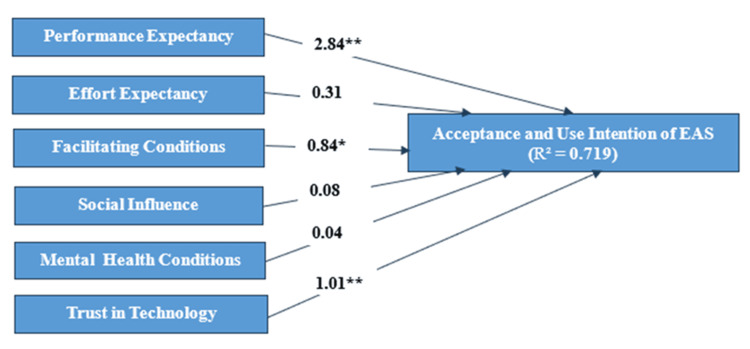
Final research model

The findings of this study support the feasibility of the UTAUT model in assessing the acceptance and use of EAS. The model showed a good fit, explaining 71.9% of the variance in the intention to use EAS (Nagelkerke R²=0.719). This suggests that the model's independent variables are highly predictive of patients' intentions to use EAS. Furthermore, the model's log-likelihood of 65.932 and the more conservative Cox & Snell R² value of 0.463 both confirm the adequacy of the model's fit, reinforcing the idea that it effectively captures the key factors influencing EAS acceptance and use. The overall model, including all predictors, is detailed in Figure 2.

## Discussion

This study primarily aimed to explore the key predictors of EAS acceptance and use among mental health patients, focusing on its reliability as a tool for accessing mental healthcare support. 

A key finding in this study is the relatively low adoption rates of the EAS, with many patients only trying it once before reverting to traditional scheduling methods, such as phone calls or in-person visits. This preference for traditional methods, as mentioned in previous studies [21,22], can be attributed to factors such as familiarity with face-to-face communication, concerns about digital literacy, and trust in the reliability of personal interactions with healthcare staff. This is particularly relevant in mental health care, where patient-provider relationships are highly valued. Patients may perceive in-person and phone-based scheduling as more reliable and personalized options. The low adoption rate of EAS within this sample highlights the need to enhance patients' digital literacy and the user-friendliness of the platforms available to encourage wider acceptance and use.

The findings indicate that even if the majority of the participants reported having attained a university or college-level education, the majority of the respondents never even tried the EAS. These results indicate that, even among educated individuals, there are challenges to the uptake of e-health services, which should be addressed in future interventions.

The findings also indicate that the highest percentage of EAS use occurred after the COVID-19 pandemic, indicating that the crisis may have accelerated the adoption of digital health services and changed healthcare access and behaviors. This aligns with previous studies [23,24], which have documented the role of the pandemic in accelerating the integration and use of digital health technologies. User satisfaction with EAS was varied, suggesting that while some users found the system beneficial, others encountered issues related to usability or effectiveness. These issues need to be addressed to improve user experience and encourage continued use.

The overall model suggests that performance expectancy, trust, and facilitating conditions positively influence the acceptance and use of EAS, while the other factors, effort expectancy, social influence, and perceived mental health conditions, don't seem to play a significant role.

The identification of performance expectancy as the key predictor of patients' intention to use EAS agrees with previous research on health technology acceptance [7,25,26]. This finding underscores the critical role of perceived usefulness in shaping user intentions. Mental health patients are more likely to use EAS when they perceive it as an effective tool for improving appointment management and enhancing their overall healthcare experiences.

Healthcare institutions should focus on showcasing the practical advantages of computerized appointment scheduling systems, particularly in terms of efficiency and effectiveness in managing appointments. Highlighting these benefits can help address patients' potential hesitations, such as concerns about usability or reliability. By prioritizing performance expectancy, healthcare providers can foster greater confidence and willingness among patients to embrace electronic services, particularly in mental health contexts.

Trust in technology emerged as a significant positive predictor of patients' intention to use EAS, indicating that individuals with higher trust in the system are more likely to engage with the service. This emphasizes the importance of trust in the process of technology acceptance, especially in healthcare contexts where privacy and reliability are paramount. These outcomes align with previous studies [19,27], which have shown that higher perceived usefulness and trust in digital mental health interventions are positively associated with increased intentions to use these interventions. This highlights the need for healthcare platforms to establish strong security measures and foster trust to encourage broader adoption.

The finding that facilitating conditions had a modest but significant positive impact on the intention to use EAS emphasizes the role of external resources, support, and infrastructure in encouraging technology adoption. Mental health patients are more inclined to accept using EAS when they perceive sufficient resources and support, which is consistent with prior research [14,28,29]. However, while facilitating conditions contribute to adoption, they are not the most influential factor in the model. Predictors like performance expectancy and trust in technology play a more substantial role in shaping patients' intentions to use EAS. This suggests that improving infrastructure and support systems is necessary but not sufficient on its own. To encourage broader EAS adoption among mental health patients, it is equally important to address psychological factors, such as demonstrating the usefulness of the technology, and practical concerns, such as ensuring trust in the platform's reliability and security. A balanced approach that combines these efforts can create a more comprehensive strategy for enhancing acceptance and sustained use of EAS.

Effort expectancy and social influence, often identified as significant predictors in some previous research [10,30], did not emerge as influential factors in this study. This aligns with findings from earlier research [15,25], suggesting that the context of mental health services may transform patient priorities. In this setting, patients appear to place greater importance on the perceived usefulness and trustworthiness of the EAS rather than its ease of use or social encouragement.

The results revealed that perceived mental health conditions did not significantly influence the intention to use EAS. This suggests that mental health patients prioritize the effectiveness and functionality of the EAS over the impact of their mental illness on decision-making. This finding underscores that the primary drivers of EAS acceptance and use are technology-focused factors, such as its efficiency and reliability, rather than emotional or psychological considerations. Consequently, strategies to improve EAS adoption should focus on enhancing its practical utility, user experience, and security features to align with patient priorities.

Limitations and future research

Although this study offers useful information about the acceptance and use of EAS, it is important to note that it also reveals several limitations, which can serve as areas for further investigation.

First, the sample size was relatively small, limiting the generalizability of the findings to larger populations. Future research should involve larger and more diverse groups, including those from rural areas or individuals with varying degrees of access to technology, to better understand the experiences and challenges across different demographics.

Second, the study primarily utilized a quantitative approach, which, while effective in identifying trends and predictors, may not capture the deeper perspectives of mental health patients. Employing qualitative methods, such as interviews or focus groups, could help uncover the specific concerns, barriers, and motivators related to EAS adoption. This approach would provide richer, context-specific insights to inform interventions.

Another limitation is the non-significance of effort expectancy and social influence, which contrasts with findings from some prior studies. Future research could explore the underlying reasons for this non-significance, such as whether these factors gain importance in different contexts, populations, or healthcare settings. For example, researchers might investigate whether effort expectancy or social influence becomes more critical for patients with low digital literacy or in communities where social networks strongly impact technology adoption.

Additionally, moderating variables like age, gender, or technological experience were not thoroughly examined in this study. These factors could play crucial roles in shaping the relationship between predictors like performance expectancy or trust and the intention to use EAS. Future studies could assess these interactions to provide more tailored and actionable insights into technology adoption processes.

## Conclusions

The study's conclusions highlight the key predictors influencing the acceptance and use of e-appointment services among mental health patients. It identified performance expectancy, trust in technology, and facilitating conditions as the most significant factors shaping patients' intentions to adopt these services. These findings emphasize the importance of ensuring that EAS platforms are perceived as effective, trustworthy, and well-supported, especially in the sensitive context of mental healthcare.

The insights gained from this study provide a valuable foundation for designing and implementing strategies aimed at promoting the widespread acceptance and use of digital mental health services. Such efforts are crucial for addressing persistent barriers to accessing quality mental healthcare. Given the potential benefits of electronic appointment systems in improving healthcare access and operational efficiency, optimizing these platforms should be a priority for healthcare providers and policymakers.
